# Can heme oxygenase-1 be a prognostic factor in patients with hepatocellular carcinoma?

**DOI:** 10.1097/MD.0000000000016084

**Published:** 2019-06-28

**Authors:** Cheon-Soo Park, Dae-Woon Eom, Yongchel Ahn, Hyuk Jai Jang, Shin Hwang, Sung-Gyu Lee

**Affiliations:** a Department of Surgery, Eunpyeong St. Mary's Hospital, College of Medicine, The Catholic University of Korea; b Department of Pathology, Gangneung Asan Hospital, University of Ulsan College of Medicine; c Department of Hematology and Oncology, Gangneung Asan Hospital, University of Ulsan College of Medicine; d Department of Surgery, Gangneung Asan Hospital, University of Ulsan College of Medicine, Gangneung; e Department of Surgery, Division of Hepatobiliary Surgery and Liver Transplantation, Asan Medical Center, University of Ulsan College of Medicine, Seoul, Republic of Korea.

**Keywords:** heme oxygenase-1, hepatocellular carcinoma, prognostic factor

## Abstract

Heme oxygenase-1 (HO-1) is an important catalytic enzyme in heme degradation, which increases during stressful conditions. It plays a major role in antioxidative and antiapoptotic processes and is associated with tumor growth and metastasis.

This study aimed to evaluate the degree of HO-1 expressions in hepatocellular carcinoma (HCC) surgical specimens and the correlation between HO-1 expression and patient prognosis. Formalin-fixed, paraffin-embedded HCC tissue samples (n = 96) were included in the analysis, and the expression of HO-1 was evaluated by immunohistochemical staining. We reviewed clinical features of patients and evaluated the prognostic role of HO-1 in patient survival and recurrence.

Positive HO-1 expression was identified in 43 cases (44.8%) and was frequently found in patients with advanced histology (Edmondson–Steiner [E-S] grade 2, 3, 4), α-fetoprotein (AFP) level of more than 200 IU/mL, and the presence of microvascular and capsular invasion (*P* < .05). In the univariate analysis, the overall survival (OS) and disease-free survival (DFS) of patients with HO-1-positive HCC were not statistically different from those with HO-1-negative HCC. Moreover, HO-1 expression was not associated with patient survival and recurrence based on the multivariate analysis. In the subgroup analysis of patients without preoperative transarterial chemoembolization (TACE) (n = 61), HO-1 was not also associated with tumor recurrence (*P* = .681).

The clinical implication of HO-1 activity is controversial in various malignancies. However, HO-1 expression did not seem to influence the prognosis of HCC patients.

## 1 Introduction

Hepatocellular carcinoma (HCC) is the most common subtype of liver cancer. Radical tumor resection is the most effective treatment, but the recurrence rate is still high and most cases undergo recurrence in the intrahepatic area.^[1]^ Unfortunately, most HCC patients suffer relapses within 2 years after operation.^[2]^ Tremendous efforts have been made to identify factors affecting patient survival, and some studies have emphasized the role of cancer cell viability, probably due to overexpression of cytoprotective proteins,^[3]^ such as inhibitors of apoptosis proteins (IAPs)^[4]^ and heme oxygenase-1 (HO-1).^[5]^

**Figure 1:**
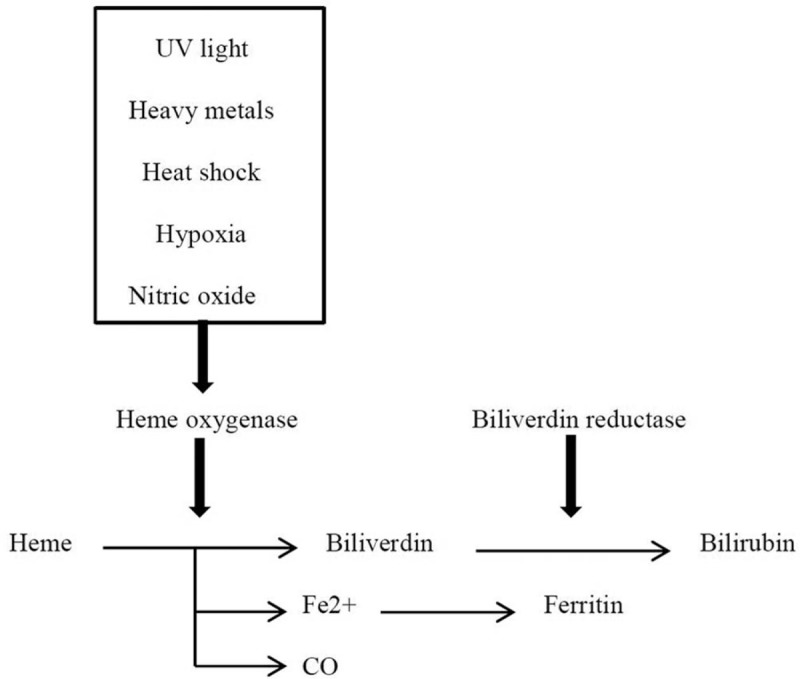
Schematic illustration of the heme oxygenase pathway. CO = carbon monoxide, Fe^2+^ = ferrous ion, UV = ultraviolet.

Accumulated evidence has supported the importance of HO-1 in cell protection against oxidative stress and other stimuli (Fig. 1).^[6]^ However, several studies have also demonstrated that HO-1 overexpression is in correlation with the pathogenesis and progression of several types of malignancies.^[7]^ In tumor-bearing mice, overexpression of HO-1 resulted in increased cell viability, proliferation, and angiogenic potential of melanoma cells and augmented distant metastasis.^[8]^ Recent studies have found that HO-1 is indirectly involved in metastasis and invasion of several types of malignancies, including breast,^[9,10]^ prostate,^[11]^ and lung cancer.^[12]^ Moreover, it has been shown that pharmacological inhibition of HO-1 activity by heavy metal derivatives, such as zinc-protoporphyrin-IX (ZnPP), induced apoptosis in hepatoma cells in vitro.^[13]^

Hence, we evaluated the degree of HO-1 expressions in the surgical specimen of HCC patients by immunohistochemical staining (IHS) and analyzed the clinical correlation between HO-1 expression and patient prognosis.

## 2 Methods

### 2.1 Patient samples

We investigated HO-1 activity in HCC tissues with the use of IHS. The clinical tissue embedded on paraffin material was obtained from 96 HCC patients who underwent curative resection from September 2005 to March 2013 at our institution. Additionally, we investigated the clinicopathologic features, recurrence, and survival of the HCC cohort. The present study protocol was reviewed and approved by the institutional review board of Gangneung Asan Hospital, University of Ulsan College of Medicine (IRB No. 2013–056), which waived the need for informed consent.

### 2.2 Immunohistochemical and H&E HO-1 staining from paraffin materials

Formalin-fixed, paraffin-embedded HCC tissue samples (n = 96) were obtained and arrayed using a tissue-arraying instrument (Quick-Ray, Unitma Co., Ltd., Seoul, Korea). Briefly, representative areas of each tumor were selected and marked on the H&E slide, and its corresponding tissue block was sampled. The designated area of each donor block was punched using a 2-mm-diameter tissue cylinder, and the sample was transferred to a recipient block. Each sample was arrayed to the duplicated blocks to minimize tissue loss.

IHS for HO-1 (Mouse monoclonal, Abcam, Cambridge, MA) was performed on the arrayed blocks. All immunostaining was performed with the Bond-Max automatic immunostaining device (Leica Biosystems, Newcastle, UK) using a bond polymer intensity detection kit (Leica Biosystems, Newcastle, UK) for formalin-fixed, paraffin-embedded tissue sections. Four-micrometer-thick sections were obtained by microtome, transferred onto adhesive slides, and dried at 62°C for 30 minutes. Antigen retrieval was carried out. Slides were counterstained with Harris hematoxylin. IHC analysis of the sections was performed without knowledge of the patient's identity or clinical status. Both the percentage of positive tumor cells and the intensity of positive staining were graded in order to obtain a semi-quantitative immunoreactive score (IRS, percentage of staining ∗ intensity of staining). The expression of HO-1 was according to the percentage of staining was graded as follows: 0, staining in < 10% of tumor cells; 1, staining in 10% to 50% of tumor cells; 2, staining in 51% to 80% of tumor cells; and 3, staining in more than 81% of tumor cells. The intensity of staining was graded as follows: 0, no or equivocal; 1, weak staining; 2, moderate staining; and 3, strong staining. Sections with IRS > 0 were all considered positive HO-1 samples. (Sections with either grade 0 in staining percentage or intensity; negative HO-1 expression)

### 2.3 Statistical analysis

For continuous variables, Student's *t* test was used for comparisons. Categorical variables were analyzed using chi-square test or Fisher exact test. Risk factors of HO-1 expression were identified by multiple logistic regression analysis. Disease-free survival (DFS) and overall survival (OS) were calculated using the Kaplan–Meier method. Prognostic factors were analyzed using the univariate Kaplan–Meier method and compared using the log-rank test to identify the predictors for survival. Multivariate regression analysis was performed using the Cox proportional hazards model to identify the independent prognostic factors for survival. A *P* value less than .05 was considered statistically significant. All statistical calculations were performed with the use of SPSS for Windows, version 19.0 (IBM Corp., Armonk, NY).

## 3 Results

### 3.1 Expression of HO-1 in patients with HCC

Positive HO-1 was verified in 43 specimens (43/96, 44.8%) by IHS. HO-1 expression tended to be found among patients with poor histological differentiation (Edmondson–Steiner [E-S] grade 2–4) (*P* = .024), presence of microvascular invasion (*P* = .038) and capsular invasion (*P* = .018), and elevated preoperative serum α-fetoprotein (AFP) (≥200 IU/mL, *P* = .025) (Table 1).

**Table 1:**
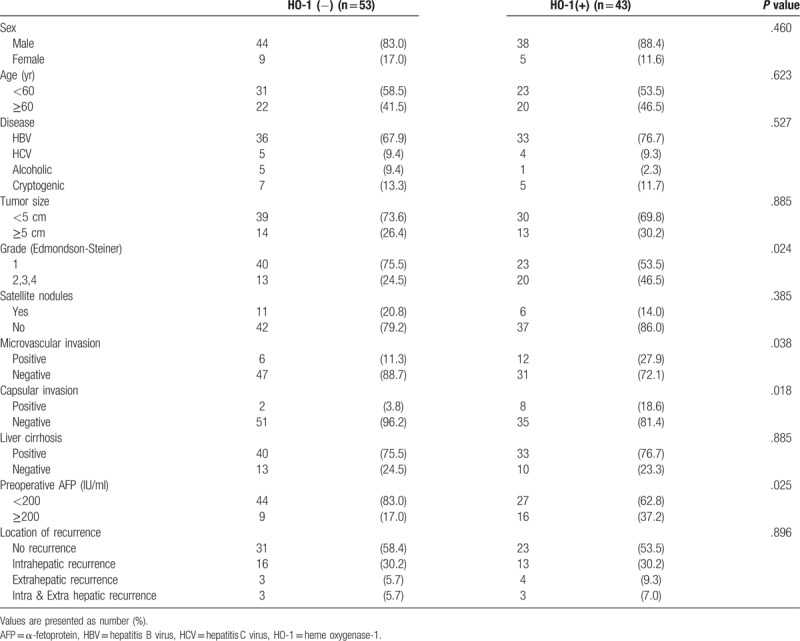
Clinicopathologic features of 96 hepatocellular carcinoma patients.

**Figure 2:**
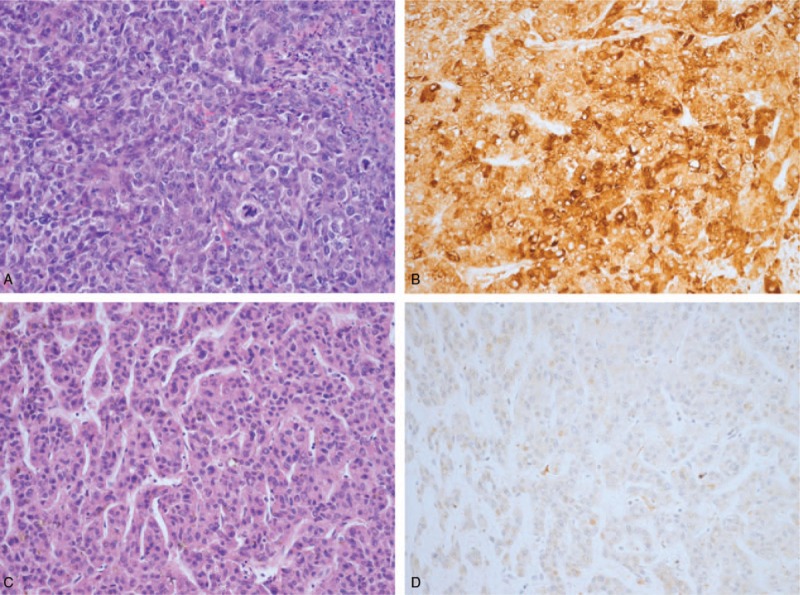
The hepatocellular carcinoma cells (A, H&E 200×) were diffusely positive for heme oxygenase-1 (B, IHC 200×). On the other hand, hepatocellular carcinoma cells (C, H&E 200×) were negative for heme oxygenase-1 expression (D, IHC 200×).

Some HCC tissues also showed diffuse HO-1 positivity in IHS (Fig. 2). In the multiple logistic regression analysis, no clinicopathologic variables were identified as risk factors of HO-1 expression in our cohorts.

### 3.2 Analysis of prognostic factors in patients with HCC

**Figure 3:**
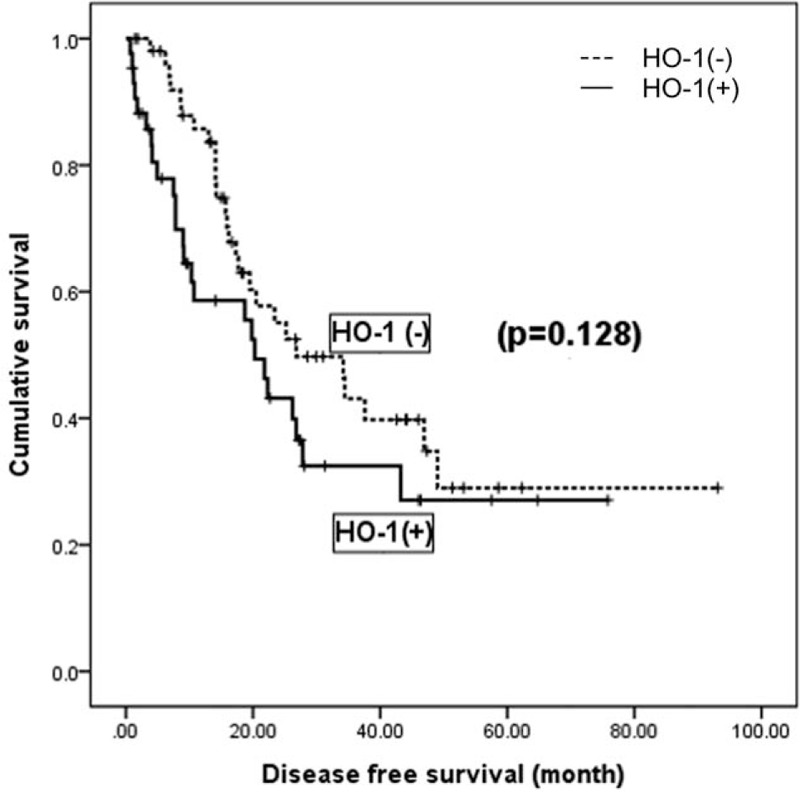
Kaplan–Meier analysis of hepatocellular carcinoma recurrence (n = 96). HO-1 = heme oxygenase-1.

In the univariate analysis (Table 2), large tumor size (≥5 cm), poor histologic grade (E-S grade 2–4), presence of capsular invasion, presence of liver cirrhosis, and high AFP (≥200 IU/mL) were found to be adverse clinical factors of recurrence (*P* < .05). Large tumor size (≥5 cm) was only an identifiable poor prognostic factor of survival. In terms of HO-1 status, OS was not affected by the presence of HO-1 (a median of 63.7 months in the positive subgroup and 64.2 in the negative subgroup, *P* = .411). There was also no statistical difference in DFS between subgroups (a median of 20.3 months in the positive subgroup and 26.8 in the negative subgroup, *P* = .128) (Fig. 3).

**Table 2:**
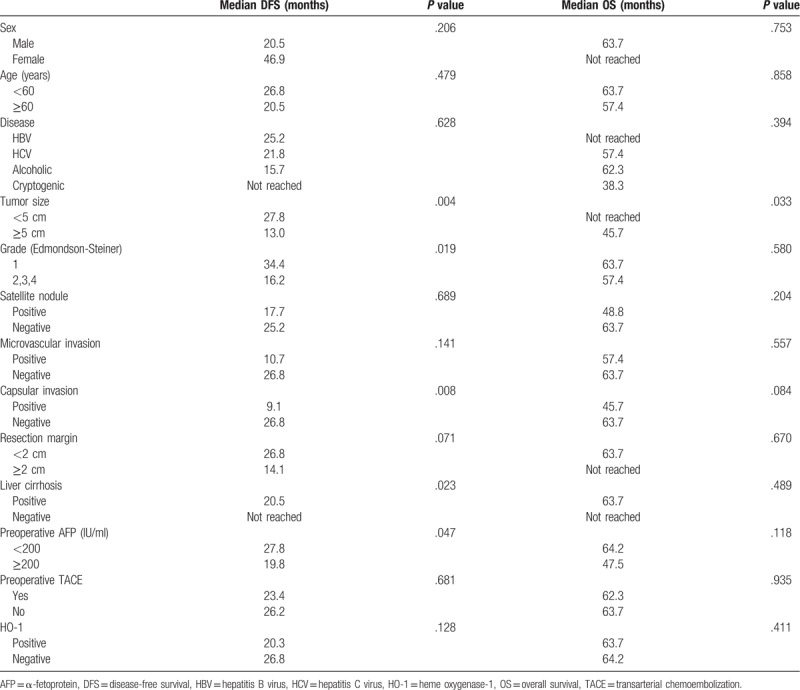
Univariate analysis of disease-free survival and overall survival in HCC patients.

**Figure 4:**
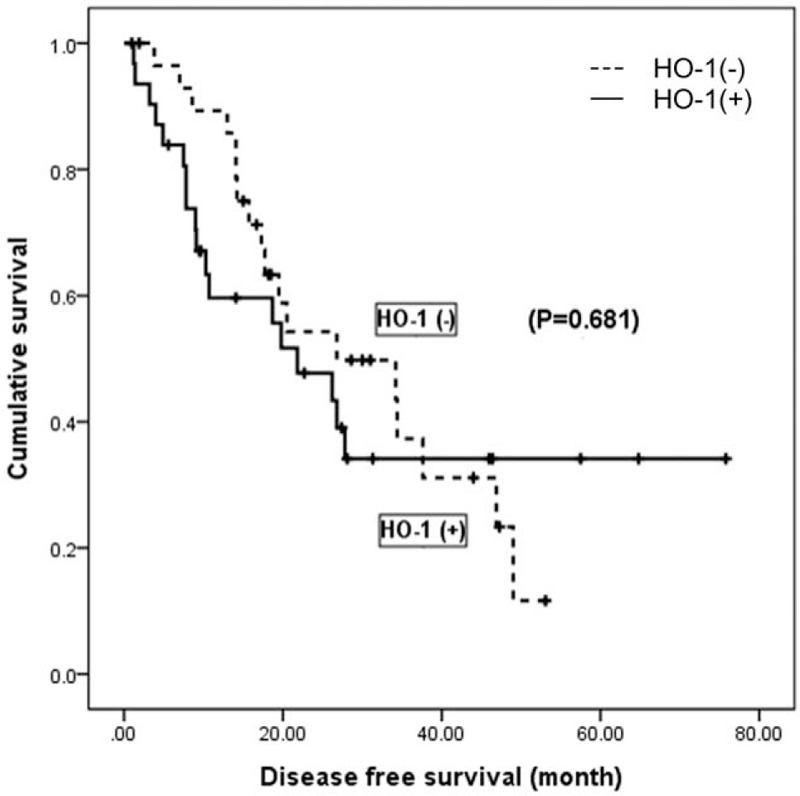
Kaplan-Meier analysis of hepatocellular carcinoma recurrence in the non-TACE group (n = 61). HO-1 = heme oxygenase-1, TACE = transarterial chemoembolization.

In the multivariable analysis (Table 3), larger tumor (≥5 cm), histologically advanced grade (E-S grade 2–4), and liver cirrhosis were statistically significant predictors of recurrence (*P* = .05). However, HO-1 expression was not associated with recurrence (*P* = .207, HR: 1.406). We presumed that preoperative transarterial chemoembolization (TACE) could affect the expression of HO-1 in HCC cells and further analyzed the effect of HO-1 expression on survival in HCC cohorts not pretreated with TACE (n = 61). There was no statistical difference between the positive and negative subgroups (*P* = .681) (Fig. 4).

**Table 3:**
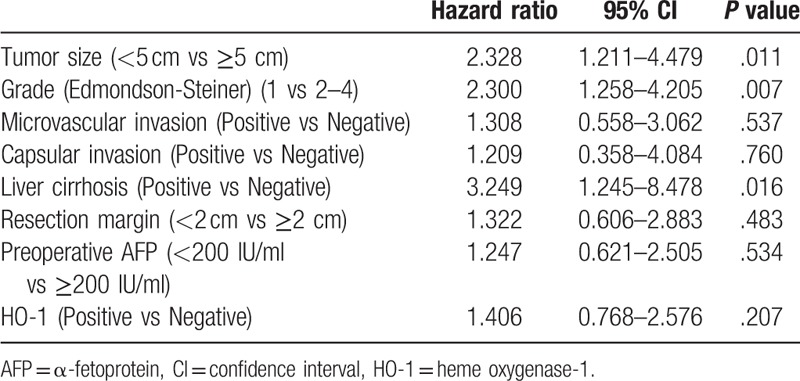
Multivariate analysis on disease-free survival in HCC patients.

## 4 Discussion

HO-1 is the rate-limiting enzyme in heme degradation. It is involved in the oxidative degradation of heme into carbon monoxide (CO), free iron, and biliverdin, which are subsequently converted to bilirubin by biliverdin reductase (Fig. 1).^[14,15]^ HO-1, also known as heat shock protein 32 (HSP 32), is an inducible isoform of HO present at low levels in most mammalian tissues. HO-1 is commonly found in both the liver and spleen. Its expression is upregulated by increased heme substrates^[16]^ and by various stimuli such as ultraviolet (UV) light,^[17]^ heavy metals,^[18]^ heat shock,^[19]^ hypoxia,^[20]^ and nitric oxide (NO)^[21]^ (Fig. 1). Increased HO-1 expression in vitro has been suggested as an adaptive process to cellular stress.^[4]^

Increased HO-1 expression has been reported in various types of human malignancy, including hepatoma, lung cancer, prostate cancer, glioblastoma, melanoma, Kaposi sarcoma, and pancreatic cancer.^[7]^ To date, many researchers have suggested that HO-1 is closely related with tumorigenesis, such as antiapoptosis, cell proliferation, invasion, and metastasis, and that it can be a potential cancer therapeutic target. Tumor cells upregulate HO-1 expression for self-protection. Tanaka et al^[13]^ reported that HO-1 plays a role in the antiapoptotic defense mechanism of tumors; increased HO-1 protects tumor cells against oxidative stress induced by NO in vivo. Some authors reported that HO-1 is related to tumor growth and metastasis in HCC. In an experimental HCC mouse model, down-modulation of HO-1 by siRNA resulted in increased cellular damage and apoptosis, thus reducing tumor growth.^[5]^ Angiogenesis is well known as a crucial step in tumor growth and is regulated by angiogenic factors such as vascular endothelial growth factor (VEGF).^[22,23]^ Furthermore, the HO-1 gene has responsive domains for many angiogenic agents and its expression induces neovascularization.^[24,25]^ HO-1 also has a stimulatory effect on VEGF expression.^[26]^

However, whether HO-1 is pro-oncogenic or otherwise is still a controversy. Some authors proposed that increased HO-1 suppressed tumor progression or migration. Lin et al^[9]^ reported that HO-1 inhibited tumor invasion by suppression of matrix metalloproteinase-1 (MMP-1) in breast cancer. Overexpression of HO-1 prevented tumor proliferation and migration in prostate cancer.^[27]^ Other in vivo and in vitro data showed that increased intracellular HO-1 proteins significantly inhibited human HCC cell migration and growth by suppressing IL-6 expression.^[28]^

To date, there have been few reports on the clinical importance of HO-1 on the basis of surgical specimens. In our study, HO-1 expression was associated with more aggressive histology. Moreover, patients with HO-1 expression tended to have preoperative higher serum AFP levels, microvascular and capsular invasion (*P* < .05) (Table 1). HO-1 expression was associated with the histological differentiation and lymph node metastasis in gastric cancer.^[29]^ In our study, logistic regression analysis identified no clinicopathologic variables as risk factors for increased HO-1 expression.

In addition, DFS and OS were not statistically significant between the positive HO-1 group and the negative HO-1 group (*P* > .05) (Table 2). Imamura et al^[2]^ reported that the pattern of HCC recurrence could be divided into early and late recurrence. Early recurrence (≤2 years) seemed to be related to tumor condition, such as microvascular invasion and satellite lesions, whereas late recurrence (≥2 years) was associated with background liver diseases and control of liver damage. In our study, 41 cases were identified as early recurrent ones. HO-1 positivity was not a predictor of early recurrence compared to cases with no recurrence (n = 44) (*P* = .478). Furthermore, we assumed that late recurrence was not affected by the presence of HO-1 at the time of operation and was more likely associated with control of background liver disease such as HBV, HCV, alcoholic LC, and nonalcoholic steatohepatitis. These findings probably resulted in no difference in DFS on the Kaplan–Meier survival curve (*P* = .128) (Fig. 3).

Doi et al^[30]^ reported that HO-1 expression in solid tumors appeared to be regulated by NO and ischemic stress. During hepatectomy, Pringle maneuver (repeated surgical procedures of clamping the portal triad for 15 minutes followed by clamp release for 5 minutes) causes ischemic damage^[31]^ and it is likely that HO-1 expression can be upregulated as a result. Since this maneuver was performed in all of our cohorts during surgery, we could presume that all the specimens were exposed to ischemic insult to a degree. To avoid this pitfall, it would be ideal to examine HO-1 expression in preoperative liver biopsy tissues. But majority of our patients received surgical resection without preoperative biopsy when HCC is strongly suspected according to patient risk factor, serologic marker, and radiographic evidences.

We looked into our data and identified 4 patients who underwent liver biopsy before surgery. Reasons for ultrasound-guided needle biopsy follow:

1. simultaneous pancreas and liver masses,
2. to distinguish HCC from intrahepatic cholangiocarcinoma,
3. new liver mass in a previous colon cancer patient,
4. to distinguish HCC from focal nodular hyperplasia.

All these patients showed HO-1 negative in the postoperative specimens. Because of very limited number of samples, we could not further analyze correlation between preoperative HO-1 expression and its impact on HCC prognosis.

Furthermore, hepatoma patients sometimes undergo TACE prior to surgery, which also causes ischemic stress. Selective arterial embolization in combination with chemotherapeutic agent infusion causes ischemic injury and acute inflammation followed by tumor necrosis. And transarterial embolization increases extrinsic apoptotic pathway activities in hepatocellular carcinoma.^[32]^ There are no experimental or clinical reports regarding an association between HO-1 expression and TACE so far. But we postulated that HO-1 expression could possibly be increased by TACE, so we performed further analysis in the non-TACE patients only in order to minimize selection bias. In the subgroup analysis for those who did not undergo TACE (n = 61), we found that the presence of HO-1 did not influence HCC recurrence (*P* = .681) (Fig. 4).

The present study has some limitations. First, the number of HCC specimens was relatively small. Second, only IHS was used for histological analysis. Finally, this study has a retrospective design. Therefore, a large multicenter prospective study using Western blotting and gene analysis should be conducted to overcome these limitations.

## 5 Conclusion

In our study, HO-1 expression in human HCC was frequently observed in patients with histologically poor grades (E-S grade 2–4), preoperatively higher AFP levels (≥200 IU/mL), microvascular and capsular invasion. However, HO-1 as a prognostic factor did not appear to play an important role in either recurrence or survival among HCC patients.

## Author contributions

**Conceptualization:** Cheon-Soo Park, Dae-Woon Eom, Yongchel Ahn, Hyuk Jai Jang.

**Data curation:** Cheon-Soo Park, Dae-Woon Eom.

**Formal analysis:** Cheon-Soo Park, Yongchel Ahn.

**Funding acquisition:** Yongchel Ahn, Hyuk Jai Jang.

**Investigation:** Cheon-Soo Park, Dae-Woon Eom, Yongchel Ahn.

**Methodology:** Hyuk Jai Jang, Shin Hwang.

**Project administration:** Yongchel Ahn, Hyuk Jai Jang.

**Resources:** Cheon-Soo Park, Dae-Woon Eom.

**Software:** Cheon-Soo Park.

**Supervision:** Shin Hwang, Sung-Gyu Lee.

**Validation:** Yongchel Ahn, Hyuk Jai Jang.

**Writing – original draft:** Cheon-Soo Park, Dae-Woon Eom.

**Writing – review & editing:** Yongchel Ahn, Hyuk Jai Jang.

Yongchel Ahn orcid: 0000-0003-0654-6664.
